# Ameliorating process parameters for zeaxanthin yield in *Arthrobacter gandavensis* MTCC 25325

**DOI:** 10.1186/s13568-020-01008-4

**Published:** 2020-04-15

**Authors:** Shristi Ram, Sushma Rani Tirkey, Madhava Anil Kumar, Sandhya Mishra

**Affiliations:** 1 Academy of Scientific and Innovative Research (AcSIR), Ghaziabad, 201 002 India; 2 Applied Phycology & Biotechnology Division, CSIR-Central Salt & Marine Chemicals Research Institute, Bhavnagar, Gujarat 364 002 India; 3 Analytical and Environmental Science Division & Centralized Instrument Facility, CSIR-Central Salt & Marine Chemicals Research Institute, Bhavnagar, 364 002 India

**Keywords:** *Arthrobacter gandavensis* MTCC 25325, Central composite design, Nutraceutical, Zeaxanthin

## Abstract

The present study aims to escalate the production of prophylactic agent zeaxanthin using a screened potential bacterial isolate. For this purpose, a freshwater bacterium capable of producing zeaxanthin was isolated from Bor Talav, Bhavnagar. The 16S rRNA sequence confirmed the isolate as *Arthrobacter gandavensis*. The bacterium was also submitted to Microbial Type Culture Collection, CSIR-Institute of Microbial Technology, Chandigarh, India, with the accession number MTCC 25325. The chemo-metric tools were employed to optimise the influencing factors such as pH, temperature, inoculum size, agitation speed, carbon source and harvest time on zeaxanthin yield. Thereafter, six parameters were narrowed down to three factors and were optimised using the central composite design (CCD) matrix. Maximum zeaxanthin (1.51 mg/g) was derived when *A. gandavensis* MTCC 25325 was grown under pH 6.0, 1.5% (w/v) glucose and 10% (v/v) inoculum size. A high regression coefficient (*R*^2^= 0.92) of the developed model indicated the accurateness of the tested parameters. To the best of our knowledge, this is the first report on tailoring the process parameters using chemo-metric optimisation for escalating the zeaxanthin production by *A. gandavensis* MTCC 25325.
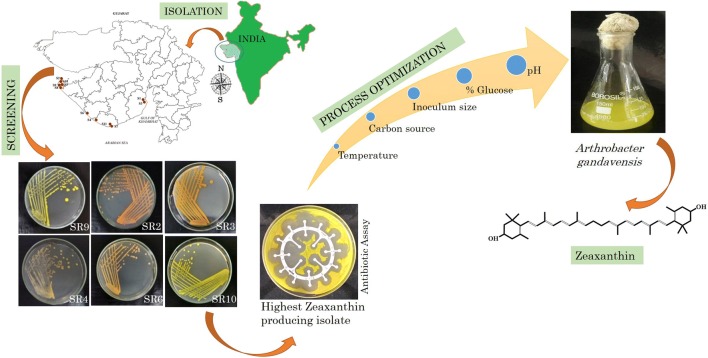

## Key points

- Chemo-metric approach was used for optimising zeaxanthin production.
- Maximum zeaxanthin was observed in the system maintained at pH 6.0, 1.5% (w/v) glucose and 10% (v/v) inoculum size.
- pH was found to be the most influencing factor dictating zeaxanthin production.
- High substrate (glucose) level inhibited zeaxanthin accumulation in *A. gandavensis* MTCC 25325.

## Introduction

The pursuit of alternative sources as functional food (that serves the dual purpose of nutrition and diet) has led to the spurred market drive for carotenoid (Numan et al. 2018a). Carotenoids reported from plant, algae, yeast and some bacteria possess high antioxidant property that circumvents cellular damages arising from oxidative stress (Freile-Pelegrín and Robledo 2013; Landete 2013). Zeaxanthin is a xanthophyll commonly found in corn, egg yolk, oranges, yellow fruits, flowers and vegetables. It imparts a yellow colour to skin and egg yolk of birds and skin colouration to swine and fish (Tibbetts 2018; Zaheer 2017) and is a well-known prophylactic agent that has been reported to exert preventive action against age-related macular degeneration and cancer (Hirahatake et al. 2019). Other application includes its use as food/feed additive and colourant (E161h) in cosmetics and food industries approved by the European Union (EU). The daily intake of zeaxanthin in diet as recommended by the Food and Nutrition Board of the Institute of Medicine’s is 0–2 mg/day (Jia et al. 2017).

Presently the demand for natural zeaxanthin is fulfilled by marigold flower (*Tagetes erecta* L), which possesses limitations such as low yields (0.3 mg/g), the season and time dependency, land use, man-power and voluminous use of irrigation water, and rigid cell walls, making round-the-year-production, a costly affair (Ram et al. 2020). Therefore, the scope of bacterial counterparts as an alternative source is now blooming for nutraceuticals production in order to overcome the low yield and high extraction costs associated with plant materials (Lee and Schmidt-Dannert 2002).

In general, Flavobacterium group is well-known not only for zeaxanthin accumulation as their main product but also act as the treasure chest for novel and rare carotenoids such as monocyclic, saproanthin and myxol (Takatani et al. 2014). They are ubiquitous organisms found widely in terrestrial, aquatic and marine environments. The first patented zeaxanthin producing bacteria belonged to this group, *Flavobacterium multivorum,* with 190 mg/L zeaxanthin content (Shepherd et al. 1976). Additionally, genetic engineering strategies employed by the researchers also assists in stimulated zeaxanthin yield. For instance, genes encoding β-C-4-oxygenase (*crtW*), the enzyme that converts β-carotene to the cyclic canthaxanthin (diketocarotenoid), have been isolated from several species of marine bacteria (Bai et al. 2017).

*Arthrobacter* sp. have been known to produce rare glucosylated C-50 carotenoid, decaprenoxanthin (Arpin et al. 1972) and reports suggesting its beneficial pharmacological activities (Numan et al. 2018b), has grasped researcher’s attention in recent years of utilising *Arthrobacter* sp., as an alternative source to the plant material, due to its competence to synthesise diverse and novel carotenoids under different cultivation conditions.

Environment mediated abiotic stressors are known to trigger carotenoid accumulation and are highly reported phenomena in the plant, algae, yeast, as well as, bacterial cells which are known for synthesising carotenoid (Xie et al. 2019). For a commercially acclaimed carotenoid source, attaining optimal conditions for maximum carotenoid production is an inevitable step.

Process optimisation through alteration of physical and chemical parameters is well-known to have a great impact on carotenoid accumulation and biomass generation. *One*-*factor*-*at*-*a*-*time* (OFAT) approach relies on changing only one factor at a time and keeping the other factors constant. This approach is simplest to implement, which primarily helps in the selection of the significant parameters affecting product yield and can serve the purpose of coarse estimation of the optimum levels (Singh et al. 2011), nonetheless, this approach can be time-consuming and laborious. However, chemo-metric tools provide a more systematic, reliable and robust method for process optimisation which has been successfully employed in several processes (Wyss et al. 2019).

In the present study, bacteria capable of producing zeaxanthin among different isolates were screened. The strains were then characterised for their biochemical and molecular identification. The physio-chemical conditions, such as harvesting time, inoculum size (v/v), pH, agitation, temperature and carbon source (as co-substrates) were altered in the highest zeaxanthin producing isolate using OFAT approach, to monitor and maximise the zeaxanthin yield (mg/g). Subsequently, the most influencing parameters were selected to further decipher the optimal production medium as well as to evaluate the interactions among different parameters empirically using central composite design (CCD).

## Materials and methods

### Media and microorganism

Glucose, beef extract, bacteriological peptone, biochemical (KB009) and antibacterial (IC002) assay kits were obtained from Hi-Media (India). All HPLC grade organic solvents like methanol, dichloromethane and acetonitrile were procured from Merck (India). Zeaxanthin standard was procured from DHI, Denmark. The bacterium used in this study, *Arthrobacter gandavensis*, is available in Microbial Type Culture Collection (MTCC), CSIR-Institute of Microbial Technology, Chandigarh with the Accession number MTCC 25325.

### Screening of potential isolates

Salt, water and soil samples were collected from 11 different sites of water bodies across Gujarat (Additional file 1: Fig. S1). Primary selection for the presence of pigment was carried out by visual detection of colour on the culture plates (Nutrient, Zobell Marine, Horikoshi and Halophilic media). The pure pigmented colonies were then cultured for 7 days in 50 mL respective broth culture. The culture was centrifuged at 10,000 rpm for 10 min, washed twice with distilled water to remove debris. The obtained pellets were subjected to carotenoid extraction by incubating the reaction mixture overnight under dark conditions using ice-cold methanol. The carotenoid production was identified using UV–Vis spectrophotometer (Varion Cary^®^500, Agilent Technology). The OD value of each sample was used to determine the carotenoid concentration using Lambert–Beer equation as described in Mishra and Singh (2010).

The methanolic extract was further analysed through Shimadzu high performance liquid chromatography (HPLC) system equipped with TSK gel ODS 120T column (Tosoh Corporation, Japan) as previously described (Ram et al. 2019) and +ESI (electrospray ionization) mode of LCMS-TOF (liquid chromatography mass spectrometry-time of flight), Waters, USA) to confirm the presence of zeaxanthin. The bacterial isolate expressing higher yield of zeaxanthin was selected and grown in nutrient broth containing (g/L): peptone, 10.0; beef extract, 10.0 and sodium chloride, 5.0.

### Identification of the potential isolate

Biochemical analyses for the isolates were performed on the Hi-Media KB009 kit, while the DNA isolation was performed using the CTAB extraction protocol mentioned in Wilson (2001). PCR was performed using 16SF-AGAGTTTGATCCTGGCTCAG and 16SR- GGTTACCTTGTTACGACTT (Mullis et al. 1986). The reaction mixture (25 µL) contained 1.0 µM of forward and reverse primer each, 100 ng of DNA, 2.5 µL of 10× Taq buffer, 0.2 mM dNTPs, 2 mM MgCl_2_ and 1 unit of Taq DNA polymerase (Fermentas, St. Leon-Rot, Germany). Thermal cycling conditions were set in Life cycler (Bio-Rad, California, USA) with an initial denaturation of 5 min of 95 °C followed by 35 cycles of denaturation at 95 °C for 45 s, annealing temperature of 60 °C for 45 s and extension temperature of 72 °C for 45 s. The final extension step was programmed for 72 °C for 6 min. The purified PCR products were subjected to Sanger dideoxy-sequencing and the phylogenetic relationship were established.

The evolutionary history was inferred by the maximum likelihood method based on the Tamura-Nei model (Tamura and Nei 1993). The heuristic search was obtained automatically by Neighbor-Join and BioNJ algorithms to a matrix of pairwise distances estimated using the maximum composite likelihood (MCL) approach and the evolutionary analyses were made using MEGA6 (Tamura et al. 2013).

### Antibiotic assay

Antibiotic sensitivity assay of the screened bacterial isolates was evaluated using a Hi-Media disc-IC002. 100 µL bacterial suspension was spread on Muller-Hinton agar plates and the standard antibiotic discs were then aseptically placed and the plates were then incubated at 37 °C for 48 h. The inhibitory activity of each antibiotic was determined by measuring the zone of inhibition.

### Inoculum preparation

Colonies of *A. gandavensis* MTCC 25325 from agar plates were inoculated into 50 mL culture nutrient broth at 40 °C, 120 rpm for 24 h for the culture to attain an exponential phase. Further, this inoculum was transferred to 1.0 L Erlenmeyer flask containing 500 mL nutrient broth and incubated overnight at 40 °C and 120 rpm. This was used as fresh inoculum for the next experiment.

### Analytical methods

On the day of harvest, two different aliquots were withdrawn, centrifuged at 10,000 rpm for 10 min and the pellet was washed twice with distilled water to remove adhered media components. The first tube containing the pellet was oven-dried with some modifications (Goswami et al. 2012) at 65 °C temperature until constant biomass was obtained. Further, for carotenoid estimation, the second tube having fresh biomass was subjected to overnight methanolic extraction under dark conditions which was then centrifuged at 10,000 rpm for 10 min. The carotenoid-containing supernatant was collected in an Amber Eppendorf tube. The carotenoid extract was stored under − 80 °C until analysed through HPLC. A gradient of the mobile phase was prepared to have; solvent A—acetonitrile:methanol:dichloromethane (80:15:5) and solvent B as acetonitrile:methanol:dichloromethane (30:20:50) (Paliwal et al. 2015). Zeaxanthin quantification was done after comparing the retention times with the standard obtained from DHI, Denmark.

### Process optimisation using OFAT approach

#### Selection of harvesting time having maximum zeaxanthin production

Effect of incubation time on zeaxanthin yield (mg/g) and dry cell weight (DCW g/L) was carried out by measuring carotenoid production at 24, 48, 72 and 96 h. Fresh culture of *A. gandavensis* MTCC 25325 having 6 × 10^6^ cfu/mL was inoculated into nutrient broth having pH 7.0 and incubated at 37 °C and 120 rpm for 96 h.

#### Effect of different *A. gandavensis* MTCC 25325 inoculum size (v/v) on zeaxanthin production

In order to check the effect of different inoculum load on the carotenoid production, the culture broth was supplemented with 2, 4, 6, 8 and 10% (v/v) inoculum size of *A. gandavensis* MTCC 25325 and incubated at 37 °C and 120 rpm. The DCW (g/L) and zeaxanthin content (mg/g) were determined after 72 h incubation.

#### Effect of different pH concentration on zeaxanthin production

The freshwater carotenoid producing bacterium, *A. gandavensis* MTCC 25325, was exposed to a wide variety of pH conditions to check the tolerance of the bacterial cell under extreme pH conditions and evaluate its carotenoid producing abilities. Different pH values were studied, such as pH 5, pH 6, pH 7, pH 8, pH 9 and pH 10 with the selected inoculum size (10% v/v) and kept for 72 h incubation at 37 °C and 120 rpm.

#### Effect of shaking condition on zeaxanthin production

To study the effect of agitation on zeaxanthin production, different agitating conditions, viz. 0 rpm (static), 60 rpm, 120 rpm and 180 rpm were studied. *A. gandavensis* MTCC 25325 was grown in nutrient broth having selected inoculum size (10% v/v) and pH 6 for 72 h.

#### Effect of different temperatures on carotenoid accumulation

The effect of different temperature regime on carotenoid accumulation, was evaluated when *A. gandavensis* MTCC 25325 was grown under 20 °C, 30 °C, 40 °C and 50 °C for 72 h. All the other optimised conditions were maintained as selected from the previous studies such as pH 6, inoculum size (10% v/v) and 120 rpm shaking conditions.

#### Effect of carbon co-substrate on zeaxanthin accumulation

Since, the nutrient broth is an undefined medium, the effect of different types of co-substrate such as glucose, sucrose and glycerol were carried out and compared to the control, which is nutrient media at a final concentration of 2% w/v. The cultivation flasks were kept in pH 6, 120 rpm shaking conditions, amended with 10% inoculum size (v/v) and 40 °C for 72 h.

### Chemometrics based optimisation

#### Batch optimisation

The different influencing production factors such as pH (5.0 to 10.0), % inoculum sizes (v/v) (2.0 to 12.0), carbon sources (glucose, fructose, sucrose and glycerol), agitation speed (0 to 180 rpm) and incubation temperatures (20 to 50 °C) were evaluated for their effect on the zeaxanthin accumulation in the selected isolate.

#### Response surface methodology (RSM)

In order to explore the interactions among the most influencing parameters, chemo-metric optimisation using CCD was proposed by ‘Design Expert’ (Version 8.0, Stat-Ease Inc., Minneapolis, USA). All experiments were conducted in triplicate using 150 mL Erlenmeyer flasks containing 50 mL of the growth medium with glucose incubated at 40 °C and 120 rpm on the basis of previously performed batch optimisation.

The statistical calculation of the coded variables in the CCD matrix is given below:

$$X_{i} = \frac{{\left( {X_{i} - X_{0} } \right)}}{\delta X}$$

where $X_{O}$, $X_{i}$ and δX denotes the coded values, center point and the step-change respectively. The chosen factors for zeaxanthin optimisation were related to the response through the following quadratic equation:

$$Y = \beta_{0} + \beta_{1} X_{1} + \beta_{2} X_{2} + \beta_{3} X_{3} + \beta_{11} X_{1}^{2} + \beta_{22} X_{2}^{2} + \beta_{33} X_{3}^{2} + \beta_{12 } X_{1 } X_{2} + \beta_{13 } X_{1 } X_{3} + \beta_{23 } X_{2} X_{3}$$

where Y is the response $$\beta_{0} ,$$$$\beta_{1}$$, $$\beta_{2}$$, $$\beta_{3}$$, $$\beta_{11, } \beta_{22,}$$$$\beta_{33}$$, $$\beta_{12}$$, $$\beta_{13}$$ and $$\beta_{23}$$ are the regression coefficients for the intercept, linear, quadratic and interaction effects respectively, $$X_{1}$$, $$X_{2}$$ and $$X_{3}$$ are the independent variables (Kumar et al. 2017). Accuracy of the model was further validated by the value of the coefficient of determination (*R*^2^), F-value, *p* value, standard error and analysis of variance (ANOVA), predicted (*R*^2^_*pre*_) and adjusted correlation coefficients (*R*^2^_*adj*_). 3D graphical representation in the form of response surface plots shows the optimum level for zeaxanthin production and reveal interactive relationships between independent parameters (Box and Draper 1987).

Further, the experimental response for various physico-chemical parameters, such as inoculum size, pH and glucose content using a face-centered design involving three levels; glucose (1.0, 1.5 and 2.0% (w/v)), pH (5.0, 6.0 and 7.0) and different inoculum sizes (10, 11 and 12% (v/v)) was fitted into a regression model involving a polynomial equation;

$$\begin{aligned} Zeaxanthin \left( {{\text{mg}}/{\text{g}}} \right) & = 25.6 + 9.67X_{1} - 10.17X_{2} + 1.08X_{3} \\ & \quad - 0.946X_{1}^{2} + 0.429X_{2}^{2} + 0.076X_{3}^{2} \\ & \quad + 0.1712X_{1} X_{2} + 0.038X_{1} X_{3} - 0.133X_{2} X_{3} . \\ \end{aligned}$$

### Statistical analysis

All experiments were performed in triplicates. The values represented were the average mean ± standard deviation (SD) of the three replicates (n = 3) obtained using Infostat software (Version 2016). All statistical significance comparisons between indicated groups were performed using a one-way ANOVA with Fisher’s post-test.

## Results

### Screening of potential bacterial strains for carotenoid production

**Fig. 1:**
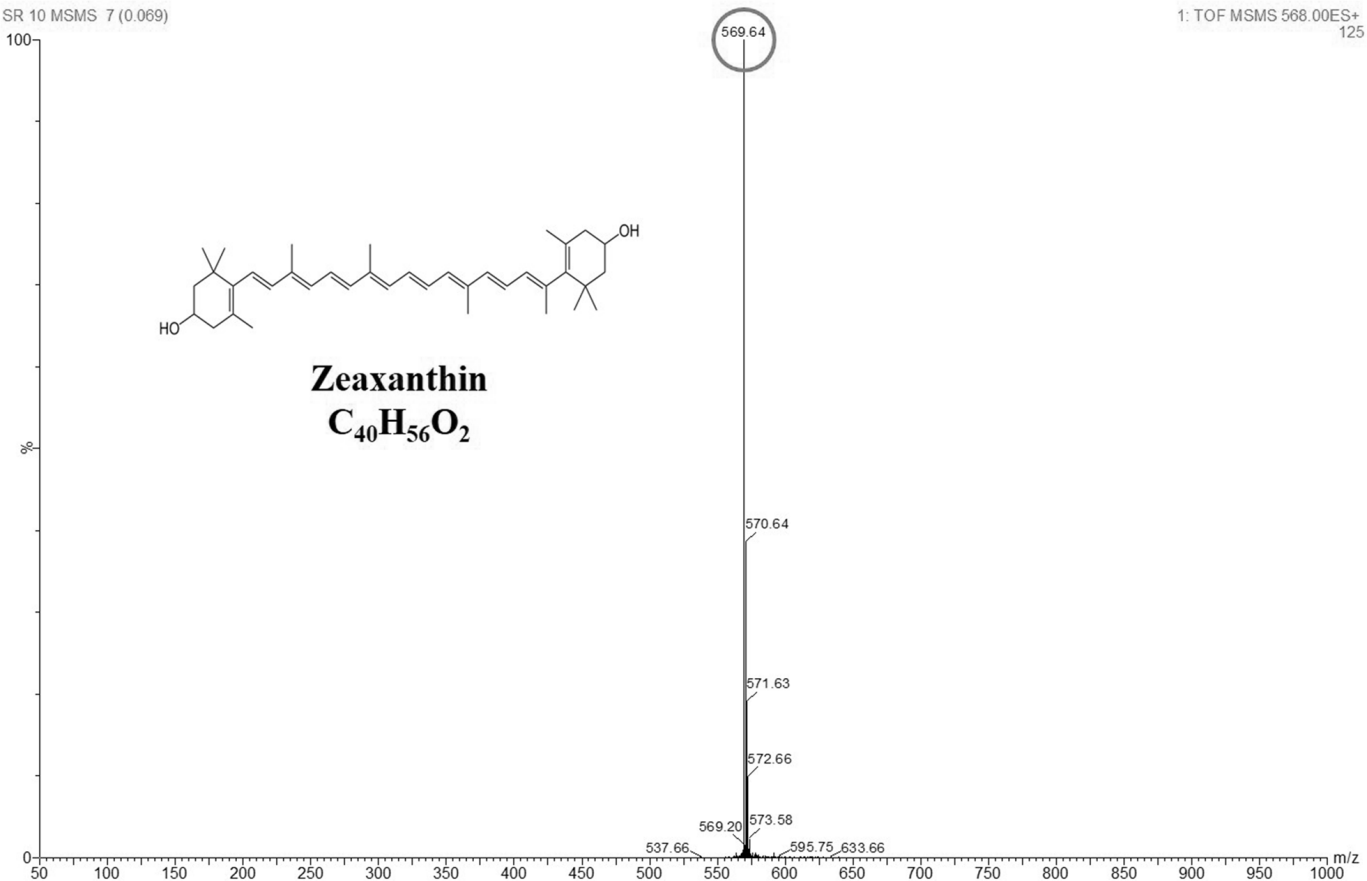
Graphs showing characteristic peaks of zeaxanthin ESI-LCMS (MS/MS) in methanolic extract of *A. gandavensis* MTCC 25325

A total of 119 pigmented bacteria were isolated, purified and screened for carotenoid production on the agar plates and HPLC analyses for 40 potential samples were done, out of which 15 isolates exerted positive carotenoid production. Methanolic extracts of the 15 screened strains were analysed using ESI–MS which confirmed the presence of the zeaxanthin peak at 568.84 m/z (Fig. 1). Biochemical assays performed on 6 carotenoid producing bacteria showed that they were able to easily utilise fructose, dextrose, maltose, xylose; moderately consume lactose, trehalose, sucrose, mannose; and was difficult in utilizing raffinose, galactose, melibiose and l-arabinose. The morphological characteristics of all the positive isolates identified them as Gram-positive.

### Molecular characterisation of the potential isolates

**Fig. 2:**
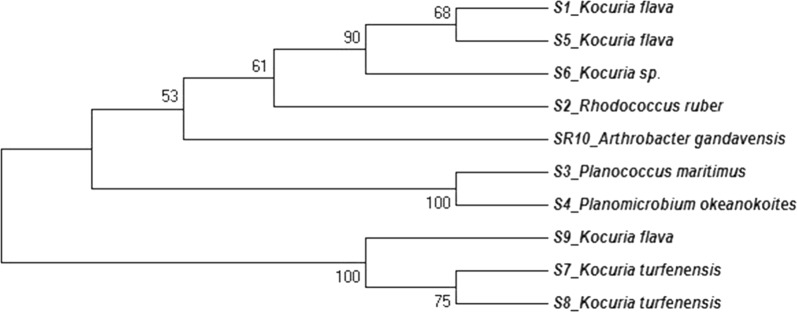
Phylogenetic tree of identified strains positive for zeaxanthin productions

The isolates identified using 16S rRNA gene sequencing were *Kocuria flava, Planomicrobium okeanokoites, Arthrobacter gandavensis* MTCC 25325*, Rhodococcus ruber*, *Planococcus maritimus* and *Kocuria* sp. (Additional file 1: Table S1). Accession numbers of the isolates submitted to NCBI are depicted in Additional file 1: Table S1. The phylogenetic relationship were established has been depicted in Fig. 2.

### Selection of the isolate for zeaxanthin production

Since, *A. gandavensis* MTCC 25325 possessed maximum total carotenoid content (234.95 µg/g) among all the potential isolates, it was selected for further study (Additional file 1: Table S1). Biochemical characterisation of *A. gandavensis* MTCC 25325 further revealed the strain was able to easily utilise lactose, xylose, maltose, fructose, dextrose, mannose, and citrate; whereas, galactose, raffinose, melibiose, l-arabinose, *O*-nitrophenyl-beta-d-galactopyranoside, esculin hydrolysis, and malonate indicated negative tests while trehalose and sucrose showed moderate utilization. Morphological determination of the selected isolate, *A. gandavensis* MTCC 25325, on agar plate suggested a round, opaque, shiny yellow colonies, having entire margin, convex elevation, moist consistency and smooth surface. The antibiotic susceptibility assays revealed *A. gandavensis* MTCC 25325 was susceptible to all antibiotics, with the highest susceptibility to azithromycin, clindamycin, chloramphenicol and linezolid as shown in Table 1.

**Table 1:** Development of various zone of inhibition (mm) exerted by the bacterial isolates on different antibiotics

Antibiotics	*Planomicrobium okeanokoites*	*Arthrobacter gandavensis*	*Kocuria flava*	*Rhodococcus ruber*	*Planococcus maritimus*	*Kocuria* sp.
Ampicillin	–	29	40	13	18	23
Clarithromycin	12	33	22	–	10	16
Gentamicin	33	25	14	23	21	33
Amoxyclav	–	23	31	19	19	30
Vancomycin	–	26	19	17	17	26
Cephalothin	–	24	40	36	34	40
Amikacin	28	21	14	22	18	33
Novobiocin	–	31	34	30	34	40
Erythromycin	10	32	34	–	–	10
Teicoplanin	–	24	25	16	16	24
Co-Trimoxazole	21	30	28	10	–	22
Penicillin	–	33	40	18	15	25
Azithromycin	32	38	37	–	22	10
Ofloxacin	20	20	20	19	24	28
Methicillin	–	26	33	37	33	37
Linezolid	–	37	31	24	25	32
Clindamycin	–	38	40	10	–	20
Tetracycline	20	28	32	11	15	40
Chloramphenicol	10	37	33	20	18	25
Oxacillin	–	16	23	26	32	39

‘–’ indicates resistant

### Process optimisation of culture conditions for zeaxanthin yield in *Arthrobacter gandavensis* MTCC 25325 using OFAT approach

#### Selection of harvesting time for maximum zeaxanthin production

**Fig. 3:**
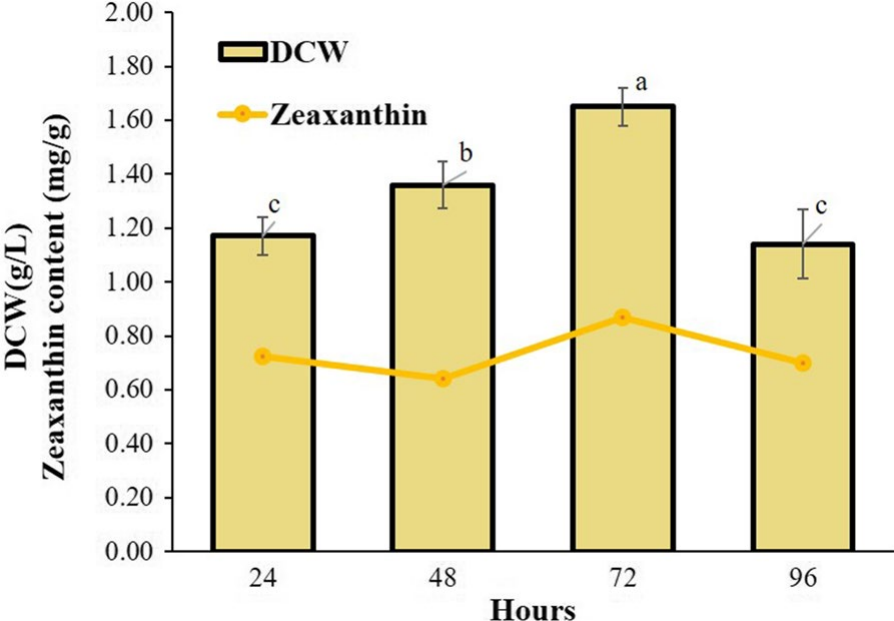
Pattern of zeaxanthin accumulation and DCW in *A. gandavensis* MTCC 25325 incubated for 96 h. All statistical significance comparisons between indicated groups were performed using a one-way ANOVA with Fisher’s post-test. Data presented are mean values and their standard deviation

Determination of the incubation time when *A. gandavensis* MTCC 25325 was cultivated in nutrient broth, revealed that zeaxanthin accumulation and DCW increases with incubation time, reaching maximum at 72 h (0.87 mg/g DCW) after which it declines (Fig. 3). Due to maximum zeaxanthin content, 72 h was selected for harvesting and carotenoid analysis in further experiments.

#### Effect of different inoculum size (v/v) on zeaxanthin production

**Fig. 4:**
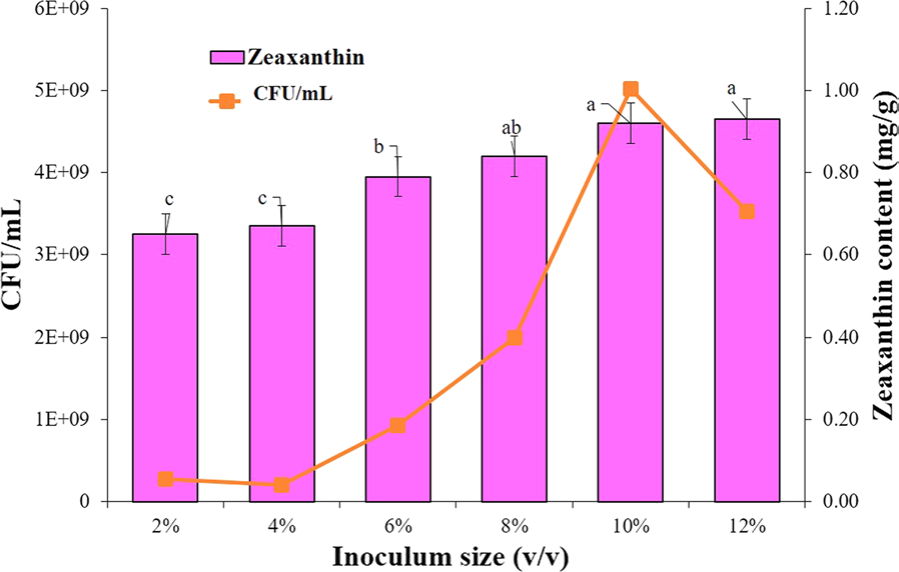
Effect of different inoculum size (v/v) on the zeaxanthin accumulation and DCW by *A. gandavensis* MTCC 25325 after 72 h. All statistical significance comparisons between indicated groups were performed using a one-way ANOVA with Fisher’s post-test. Data presented are mean values and their standard deviation

The data suggests that inoculum load is directly proportional to zeaxanthin accumulation and reaches maximum at 10 and 12% (v/v) inoculum size. The lowest carotenoid formation was observed when 2% inoculum size was added (0.65 mg/g DCW). The addition of 10% inoculum size yielded 1.4-fold higher zeaxanthin content (0.92 mg/g DCW). As zeaxanthin content were significantly similar for 10 and 12% (v/v) inoculum sizes, hence subsequent optimisation studies were carried out using 10% (v/v) inoculum size (Fig. 4).

#### Effect of different pH concentration on zeaxanthin production

**Fig. 5:**
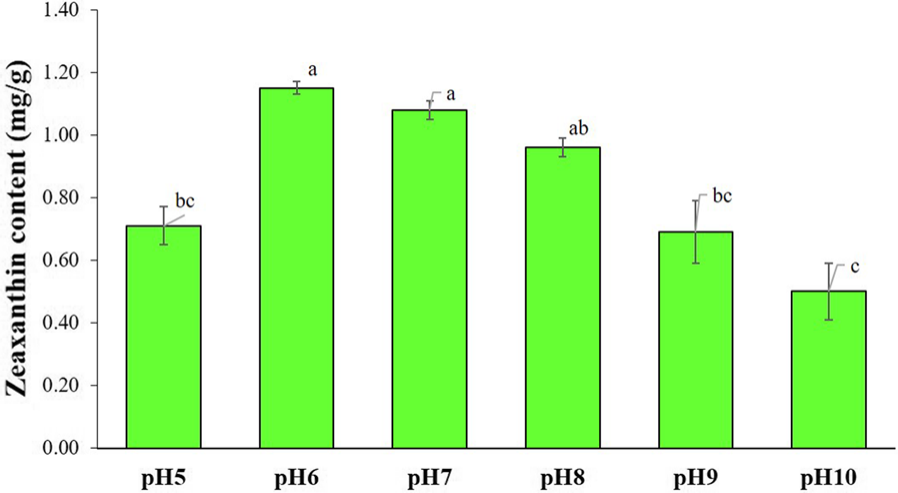
Effect of different culture pH the zeaxanthin accumulation by *A. gandavensis* MTCC 25325 after 72 h. All statistical significance comparisons between indicated groups were performed using a one-way ANOVA with Fisher’s post-test. Data presented are mean values and their standard deviation

In this study, both acidic and alkaline conditions were considered as inhibitory for carotenoid production. The highest zeaxanthin yield was found to be at pH 6 (1.15 ± 0.02 mg/g DCW) and pH 7 (1.08 ± 0.03 mg/g DCW). However, further increase in pH value had inhibitory effect on zeaxanthin content, and the lowest carotenoid was recorded in pH 10 (0.50 ± 0.09 mg/g DCW). Therefore, pH 6 was selected for further studies (Fig. 5).

#### Effect of shaking condition on zeaxanthin production

**Fig. 6:**
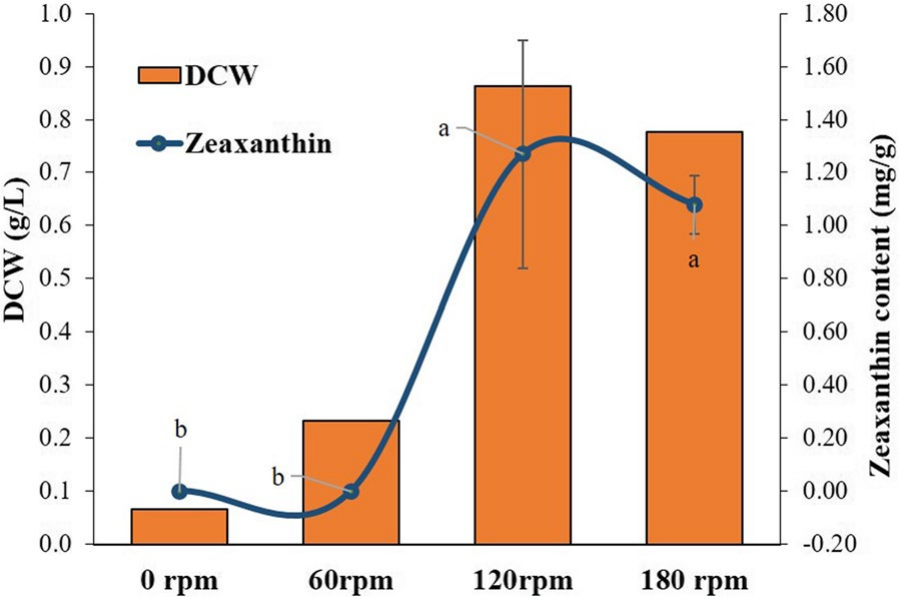
Effect of shaking conditions on the zeaxanthin accumulation by *A. gandavensis* MTCC 25325 after 72 h of incubation. All statistical significance comparisons between indicated groups were performed using a one-way ANOVA with Fisher’s post-test. Data presented are mean values and their standard deviation

Aeration was found to be an indispensable factor for the carotenoid synthesis in *A. gandavensis* MTCC 25325, as there was lack of zeaxanthin production under static condition; whereas maximum carotenoid content was observed when culture was shaken at 120 (1.27 mg/g DCW) and 180 rpm (1.08 mg/g DCW). Figure 6 shows a close relationship between the DCW and zeaxanthin content, a steady rise in the zeaxanthin accumulation was observed with the increasing rpm. As no significant difference was obtained in 120 and 180 rpm, 120 rpm was selected for further study as it consumes less electrical power.

#### Effect of different temperatures on zeaxanthin production

**Fig. 7:**
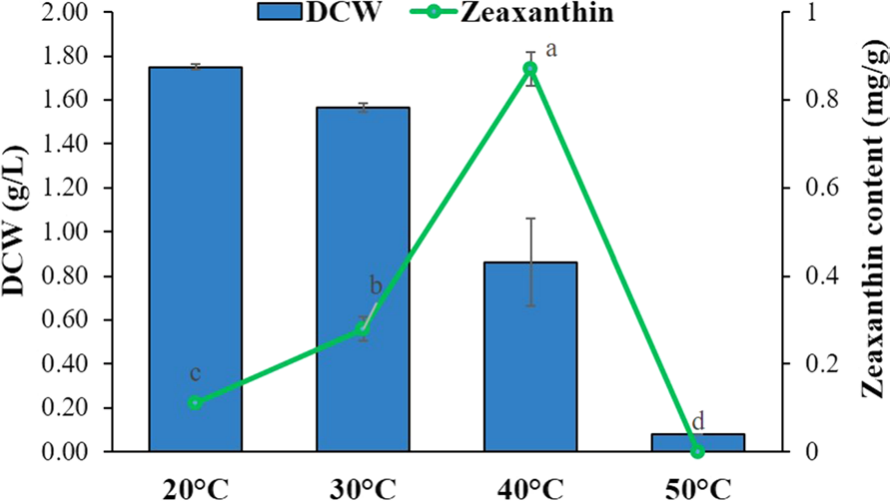
Effect of various temperature conditions on *A. gandavensis* MTCC 25325 zeaxanthin accumulation after 72 h of incubation. All statistical significance comparisons between indicated groups were performed using a one-way ANOVA with Fisher’s post-test. Data presented are mean values and their standard deviation

As shown in Fig. 7, temperature governed the DCW and zeaxanthin content of *A. gandavensis* MTCC 25325. Though a moderate temperature of 20 °C was found to improve DCW (1.75 g/L), it was not found favourable for carotenoid production (0.11 mg/g DCW). Interestingly, although temperature was found to be inversely proportional to the DCW, zeaxanthin content increased by ~ eightfold at 40 °C (0.87 mg/g DCW) after which it drastically declined due to the lack of growth (Fig. 7).

#### Effect of carbon co-substrate on zeaxanthin accumulation

**Fig. 8:**
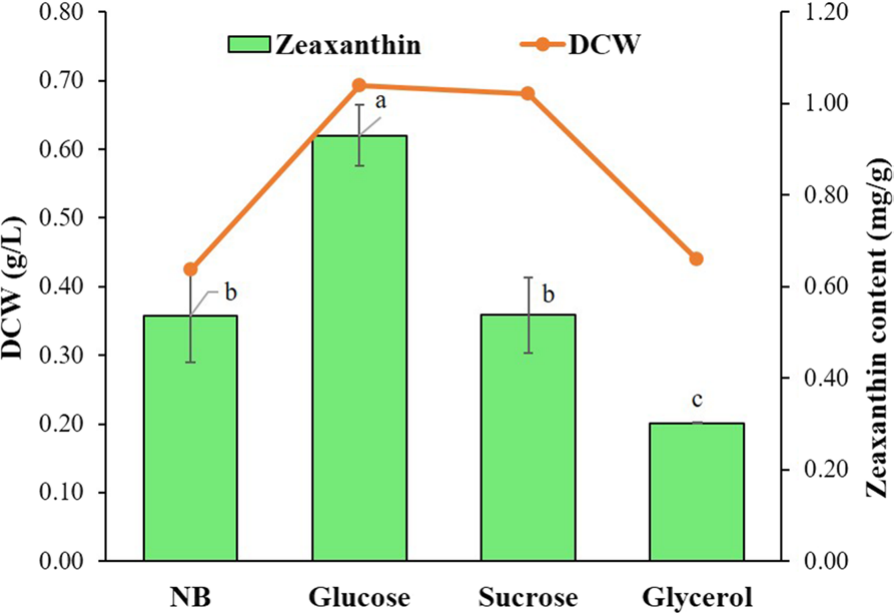
Effect of different carbon source as co-substrate for zeaxanthin accumulation by *A. gandavensis* MTCC 25325 after 72 h. All statistical significance comparisons between indicated groups were performed using a one-way ANOVA with Fisher’s post-test. Data presented are mean values and their standard deviation

The result showed that addition of glucose as co-substrate significantly improved the DCW and zeaxanthin content in *A. gandavensis* MTCC 25325. The highest zeaxanthin content of 0.93 mg/g was achieved when grown in the medium supplemented with glucose, ~ 1.7-fold increase as compared to the control, while the least content of zeaxanthin was noted in glycerol (0.30 mg/g DCW). Zeaxanthin content in sucrose was similar to control however, significant increase in the dry cell weight was observed when grown in the presence of sucrose as compared to the control (Fig. 8).

### Production of zeaxanthin using *A. gandavensis* MTCC 25325 using CCD

The order of the pH on the zeaxanthin yield followed pH 5.0 < pH7.0 < pH 6.0. Further, it could be seen that pH 7.0 supported low zeaxanthin yield (0.02 mg/g), while at < pH 5.0 zeaxanthin production was completely retarded. The highest zeaxanthin yield was found to be 1.51 mg/g at pH 6.0. The result suggested that optimised growth medium for *A. gandavensis* MTCC 25325 for maximum zeaxanthin production was observed when cultivated under pH 6.0, 1.5% (w/v) glucose concentration and 10% inoculum when harvested at 72 h.

**Fig. 9:**
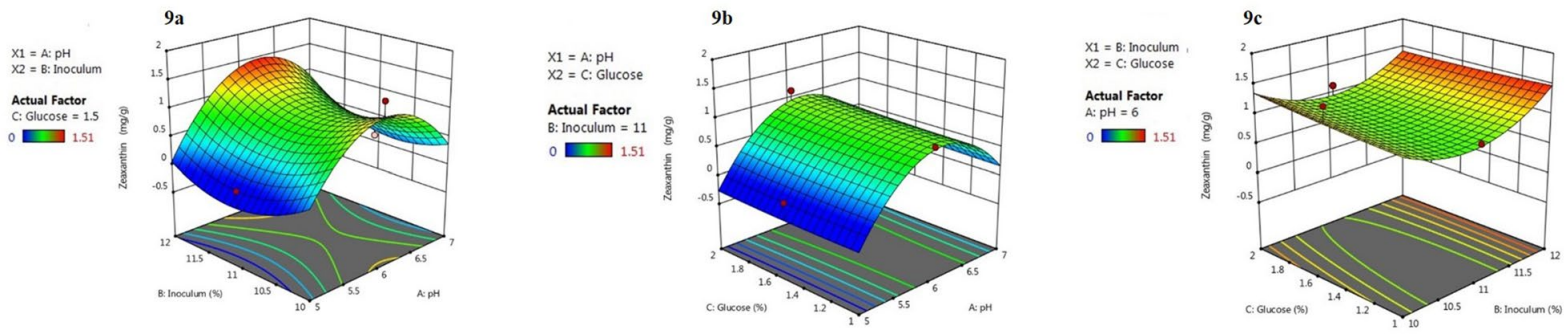
**a** Response surface plot of zeaxanthin yield vs inoculum, pH. The regions of surface plot are divided in three regions depicting zeaxanthin yield in mg/g, glucose concentration, and inoculum size in percentages. **b** Yield vs glucose, inoculum, and **c** yield vs glucose, pH

On the contrary, inoculum size (%v/v) is not influential in zeaxanthin production (Fig. 9c). Additionally, the glucose concentration had both synergistic as well as the antagonistic effect on the zeaxanthin yield. The reason for low zeaxanthin production at higher glucose concentrations may be attributed to substrate repression (Goswami et al. 2012). Our study further corroborates with Goswami et al. (2012) where a significant decrease in canthaxanthin from *Dietzia maris* was observed at glucose concentration higher than 1.5%. In another study, glucose concentration (> 0.75%) declined the β-cryptoxanthin yield from *Kocuria marina* (Mitra et al. 2017). Conclusively, the predicted optimised process parameter for yielding higher zeaxanthin from *A. gandavensis* MTCC 25325 would be achieved at pH 6.0, 10% inoculum and 1.5% (w/v) glucose. *A. gandavensis* MTCC 25325 is a freshwater isolate, therefore it is able to grow well near neutral pH conditions, however, when the pH of the culture media is shifted towards acidic conditions, it undergoes abiotic stress eliciting higher amount of zeaxanthin.

## Discussion

On the basis of the highest total carotenoid content (234.95 µg/g) in *A. gandavensis* MTCC 25325 among all the tested isolates, this strain was investigated for higher zeaxanthin yield employing process optimisation strategies.

### Process optimisation of culture conditions for zeaxanthin yield in *A. gandavensis* MTCC 25325 using OFAT approach

*A. gandavensis* MTCC 25325 was found to accumulate highest zeaxanthin at 72 h incubation in culture media. A similar result was obtained with *Muricauda* sp. (Prabhu et al. 2013) and *Siansivirga zeaxanthinifaciens* (Hameed et al. 2012); at 72 h of cultivation, these species showed highest zeaxanthin yield of 1.02 mg/g and 6.5 mg/g, respectively. However, harvesting time may vary widely from species to species. For instance, the maximum zeaxanthin yield was 44 h for *Flavobacterium multivorum* (3.74 mg/L) (Bhosale et al. 2004).

A direct correlation between inoculum size (% v/v) and zeaxanthin accumulation was observed. Highest zeaxanthin content was observed by 10 and 12% inoculum size, this may be due to the rapid utilisation of nutrients from the culture media at high inoculum load, which ensured rapid reach of the stationary phase, thereby quick carotenoid accumulation (Sandhya et al. 2005).

pH is one of the most crucial factor for supporting the growth of bacteria as it helps in maintaining the osmotic pressure of bacterial cellular membranes. Change in pH triggers changes in the osmotic potential of the cell, required to maintain its integrity. Much deviation from the optimum pH results in its rupture because of turgor pressure. pH 6 and pH 7 supported maximum carotenoid yield, these results are in congruence with the previous reports on bacterial zeaxanthin production (Thawornwiriyanun et al. 2012). *Sphingomonas natatorial* showed the highest zeaxanthin yield at pH 7 howbeit maximum DCW was attained at pH 6 (Thawornwiriyanun et al. 2012). Whereas, for *Muricauda* sp., both optimal growth and zeaxanthin production were achieved at pH 7 (Prabhu et al. 2013).

In aerobic bacteria, aeration had a better impact on zeaxanthin production, as utilization of oxygen occurs in various carotenoid biosynthesis steps, viz. cyclisation, desaturation and oxygenation (Sowmya and Sachindra 2015). Gradual increase was observed in zeaxanthin accumulation as the agitation was increased reaching maximum at 120 and 180 rpm, bacterial carotenoid accumulation and DCW increased when incubated under shaking conditions. Shaking condition provides aeration to the bacteria which stimulates, the biomass generation of aerobic bacteria as it mixes the nutrient and makes them bioavailable and aerates the solution (Juergensmeyer et al. 2007).

Zeaxanthin forms an integral component of cellular membranes in bacteria, thereby assisting in photo protection, rigidity and regulating membrane fluidity (Ram et al. 2020). In the studied mesophile, *A. gandavensis* MTCC 25325, a higher temperature (40 °C) was found to stimulate carotenogenesis, however, lower temperatures encouraged biomass production. The stimulatory effect of temperature on carotenogenesis has been explained by hypothesising reduction in the efficiency of secondary metabolic reactions (the origination of coloured carotenoids) at lower temperatures (Avalos et al. 2017). However, as per the available reports the optimum temperature for zeaxanthin production varies within species. For example, optimum growth and zeaxanthin production for *Muricauda lutaonensis* was observed at 40 °C (Hameed et al. 2011) whereas for *Muricauda* sp. and *Muricauda olearia* the optimum temperature was 32 °C (Prabhu et al. 2013). Elsewhere, Thawornwiriyanun et al. (2012) recorded 30 °C as an ideal temperature for zeaxanthin production in *Sphingomonas natoria*. In a recent study, after 192 h of thermal stress at 31.6 °C, *Muricauda lutaonensis* (a *Flavobacteriaceae* bacteria) showed zeaxanthin yield of 8.04 × 10^−2^ µg/mL when grown in association with a coral *Galaxea fascicularis* and an alga *Symbiodiniaceae* (Motone et al. 2020). They have suggested that carotenogenesis in *M. lutaonensis* was governed by oxidative stress and also mentioned that zeaxanthin produced from this bacterium helps the alga and coral holobiont in mitigating environmental stress by reducing the production of reactive oxygen species (ROS).

Carbon sources are required to create carbon flux for energy generation and general metabolism. Since the choice of substrate utilisation is species specific, there is a need to optimise physico-chemical parameters for efficient production of desired product. Addition of glucose as co-substrate significantly improved the zeaxanthin content (0.93 mg/g), while least was observed in the addition of glycerol. In a similar work reported by Sowmya and Sachindra (2015), a significant improved carotenoid content was achieved in control (0.70 mg/L) followed by glucose (0.66 mg/L). This is further corroborated in *Flavobacterium* sp., as although the supplementation of glucose elicited carotenoid production, maximum growth was achieved with sucrose (Alcantara and Sanchez 1999). High productivity of zeaxanthin in presence of glucose signifies the organism’s ability to metabolise glucose and its utilisation as precursor in carotenoid biosynthesis. In order to ascertain the route of isoprenoid precursor in a zeaxanthin producing strain, *Paracoccus* sp. PTA 3335 was radio labelled (^13^C) glucose was used. The trailing of this radiolabelled glucose disclosed its utilisation through Embden–Meyerhof–Parnas and mevalonate pathways prior biosynthesis of the isoprenoid precursors (Eisenreich et al. 2002). Previously, Morris (1960) showed that unlike *Paracoccus* sp., glucose metabolism in *Arthrobacter globiformis* takes place via EMP pathway and hexose monophosphate shunt. Radio-labelled glucose molecule used by Morris confirmed that the end product of glucose metabolism pathway i.e. pyruvate was further oxidized in tricarboxylic acid cycle (TCA) resulting in formation of acetyl co-A used in zeaxanthin biosynthesis in MVP. Based on these previous reports, it is evident that glucose metabolism plays an essential role in the zeaxanthin biosynthesis pathway thus supporting the role of glucose in ameliorating zeaxanthin content of *A. gandavensis* MTCC 25325 in the current study. In conclusion, among all the tested bacterial isolates, *A. gandavensis* MTCC 25325 showed the highest carotenoid content and the batch optimisation trails revealed that the zeaxanthin production was high in the glucose supplemented production medium with pH 6.0, 10% (v/v) inoculum size, 120 rpm, on the 72 h of harvest and at 40 °C temperature.

### Production of zeaxanthin in *A. gandavensis* MTCC 25325 using CCD

Optimisation of inoculum volume, pH and glucose content was performed and the zeaxanthin yield against the different combinations of the parameters is shown in Table 2. The model validation was checked using analysis of variance (ANOVA) and the value of *R*^2^ was 0.928 for zeaxanthin production which signifies that the statistical model explained with 92.8% accuracy, the value for variability in different experiments. Subsequently, the F-value (14.46) of the model is several times higher than the *p*-value (Table 3), further inferring that the obtained regression model was highly significant (Pathak et al. 2015). The optimum operational parameters for zeaxanthin production using *A. gandavensis* MTCC 25325 is easily interpreted using the obtained response surface plots (Fig. 9a–c). Low *p*-value (> 0.05) and squared terms dictate the hump-ness of the curve, revealing a significant independent variable (Table 3).

**Table 2:** CCD matrix showing the zeaxanthin yield on 3rd day, agitation at 120 rpm and 40 °C using *A. gandavensis*

Run	pH	Inoculum (%)	Glucose (%)	Zeaxanthin (mg/g)
1	5.0	10	1.0	0.07
2	7.0	10	1.0	0.22
3	5.0	12	1.0	0.06
4	7.0	12	1.0	1.07
5	5.0	10	2.0	0.12
6	7.0	10	2.0	0.52
7	5.0	12	2.0	0.02
8	7.0	12	2.0	0.93
9	5.0	11	1.5	0.00
10	7.0	11	1.5	0.12
11	6.0	10	1.5	1.51
12	6.0	12	1.5	1.36
13	6.0	11	1.0	0.92
14	6.0	11	2.0	1.13
15	6.0	11	1.5	0.83
16	6.0	11	1.5	0.83
17	6.0	11	1.5	0.83
18	6.0	11	1.5	0.83
19	6.0	11	1.5	0.83
20	6.0	11	1.5	0.83

**Table 3:** ANOVA for response surface polynomial equation and corresponding F-values and *p*-values for zeaxanthin production by *A. gandavensis*

Source	Degree of freedom	Adj SS	Adj MS	F-value	P-value
Model	9	3.95944	0.43994	14.46	0.000
Linear	3	0.78525	0.26175	8.60	0.004
X_1_	1	0.67081	0.67081	22.05	0.001
X_2_	1	0.10000	0.10000	3.29	0.100
X_3_	1	0.01444	0.01444	0.47	0.507
Square	3	2.90165	0.96722	31.79	0.000
*X*_1_^2^	1	2.46055	2.46055	80.88	0.000
*X*_2_^2^	1	0.50633	0.50633	16.64	0.002
*X*_3_^2^	1	0.00100	0.00100	0.03	0.860
2-way interaction	3	0.27254	0.09085	2.99	0.083
*X*_1_*X*_2_	1	0.23461	0.23461	7.71	0.020
*X*_1_*X*_3_	1	0.00281	0.00281	0.09	0.767
*X*_2_*X*_3_	1	0.03511	0.03511	1.15	0.308
Error	10	0.30422	0.03042		
Lack-of-fit	5	0.30422	0.06084		
Pure error	5	0.00000	0.00000		
Total	19	4.26365			
*R*^2^	0.928				

The plots imply the behavior of response (zeaxanthin production) against the influence of three independent variables and are depicted in Fig. 9a–c. The curve of the response surface plot provides a means to visually interpret the interactions amongst the independent variables. The central hump on the response surface plot aid to deduce the optimum condition resulting from interactions amongst the variables and the output. An ellipsoid curve showcase significant interaction between the independent variable and the response (Muralidhar et al. 2001).

Intracellular accumulation of zeaxanthin in response to variation in culture conditions in *Flavobacterium multivorum* (Bhosale et al. 2004) or carotenoid elicitation due to the reactive oxygen species mediated oxidative stress in microbes has been a popular strategy (Bhosale 2004). Maintaining the pH of the fermentation broth is a vital parameter as the metabolite production hinges on it. A further shift towards the acidic environment, the isolate is unable to survive as its cell membrane composition is compromised as a response towards low pH. Here, pH has been proved to be the most significant parameter amongst all as shown in Fig. 9a, b. Thus maximum zeaxanthin production was observed when *A. gandavensis* MTCC 25325 were exposed to low pH (pH 6.0). Similar results were obtained on *Rhodotorula cheniorum* (Nasrabadi and Razavi 2011) and *Serratia marcescens* (Wang et al. 2012) at pH 5.85 and pH 6.0 respectively. The two dimensional (2D) plots also helped to understand that the most significant parameter is pH 6.0 (represented as the darkest green) and further to it, the colour lightens.

The implementation of chemo-metric tools for process optimisation seemed to be a prudent strategy which further evinced that pH and substrate utilization influences the greater accumulation of zeaxanthin in *A. gandavensis* MTCC 25325 (1.51 mg/g). The outcome of this study further establishes the fact that abiotic stress is a potent carotenogenesis elicitor. The interpretation of the significant and non-significant parameters using design of experiments aided visual interpretation. Thus the outcome of this study could be useful in providing guidelines for selecting the optimal conditions for zeaxanthin production.

The study demonstrates that abiotic factors highly influences the growth and carotenoid accumulation in *A. gandavensis* MTCC 25325. Other physico-chemical factors employed for the study altered the growth media were also found to greatly affect the metabolism and carotenoid production ability. The carotenoid yield increased with an increase in the incubation time, reaching maximum at the 3rd day of incubation. The pH of the growth media had a significant effect on the yield of carotenoids with a better yield at pH 6. Agitation stimulated zeaxanthin production and 120 rpm was found to be best for its cultivation. Similarly, 40 °C was found to accumulate higher zeaxanthin content than 30 °C and 20 °C, whereas there was no growth observed at 50 °C. The addition of glucose as a co-substrate significantly improved zeaxanthin production. Furthermore, the RSM validated the model and showed that the optimised parameters for higher zeaxanthin production are pH 6, 10% (v/v) inoculum and 1.5% (w/v) glucose content. The interaction between different parameters further, suggests that pH was the most influential parameter as it gave a hinged contour plot. The marginal difference observed with respect to DCW and zeaxanthin content in different sets of experiments can be attributed to the difference in the inoculum load.

## Supplementary information

**Additional file 1. Table S1.** General characteristics of bacterial isolates showing zeaxanthin production. **Fig. S1.** Sampling location of pigmented bacterial isolates screened for zeaxanthin production.

## Data Availability

Corresponding author could provide the all experimental data on valid request.
