# Supplementary material for: Ameliorating process parameters for zeaxanthin yield in Arthrobacter gandavensis MTCC 25325
Source: AMB Express. 2020 Apr 15;10:69. doi: 10.1186/s13568-020-01008-4 (PMC7158978; doi:10.1186/s13568-020-01008-4)
Supplement: Supplementary file 1 — Additional file 1. Table S1. General characteristics of bacterial isolates showing zeaxanthin production. Fig. S1. Sampling location of pigmented bacterial isolates screened for zeaxanthin production. [file 13568_2020_1008_MOESM1_ESM.pdf]

Journal name: AMB Express

Ameliorating process parameters for zeaxanthin yield in *Arthrobacter gandavensis* MTCC 25325

Shristi Ram[a,b], Sushma Rani Tirkey[a,b], Madhava Anil Kumar[a,c], Sandhya Mishra[a,b]*

[a]Academy of Scientific and Innovative Research (AcSIR), Ghaziabad-201 002, India.

[b]Applied Phycology & Biotechnology Division, CSIR-Central Salt & Marine Chemicals Research Institute, Bhavnagar – 364 002, India.

[c]Analytical and Environmental Science Division & Centralized Instrument Facility, CSIR-Central Salt & Marine Chemicals Research Institute, Bhavnagar-364 002, India .

*Author for correspondence

Dr. Sandhya Mishra

Emeritus Scientist

Applied Phycology & Biotechnology Division

CSIR-Central Salt & Marine Chemicals Research Institute

Bhavnagar-364 002, Gujarat, India

Tel: +91 278 256 5801 / 256 3805 Extn. 6160

Fax: + 91 278 256 6970 / 256 7562

Email address: smishra@csmcri.res.in

ORCID iD: 0000-0002-2412-4927

Additional File

Table S1 General characteristics of bacterial isolates showing zeaxanthin production

**Isolate name**	**Gram staining**	**Sampling site**	**16s rRNA gene sequencing**	**Total carotenoid content (μg/g)**	**Accession number**
SR9	+	CSMCRI-ESF	*Kocuria flava*	85.11	--
SR2	+	Somnath	*Rhodococcus ruber*	37.96	--
SR3	+	Okha road	*Planococcus maritimus*	158.92	*MF276799*
SR4	+	CSMCRI-Experimental Salt Farm	*Planomicrobium okeanokoites*	75.48	*MF276800*
SR6	+	Gangeshwar	*Kocuria* sp.	136.06	*MF276789*
SR10	+	Bor-Talav	*Arthrobacter gandavensis*	234.95	*MH544097.1*

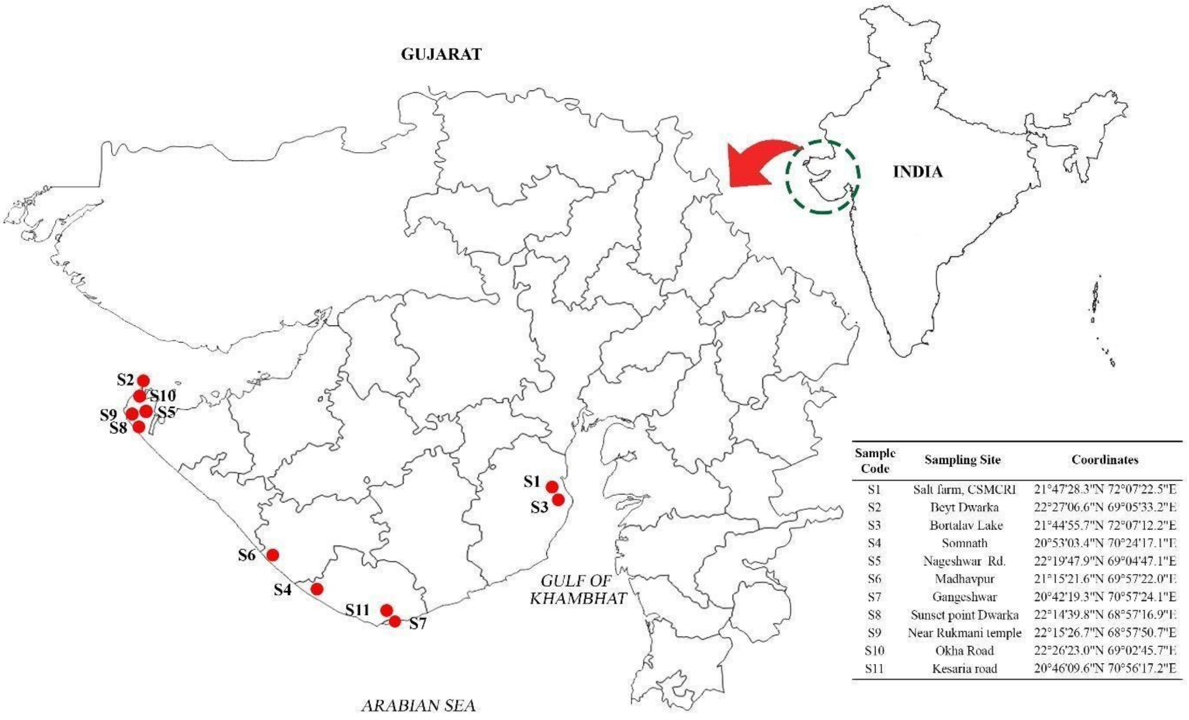

Sample Code	Sampling Site	Coordinates
S1	Salt farm, CSMCRI	21°47'28.3"N 72°07'22.5"E
S2	Beyt Dwarka	22°27'06.6"N 69°05'33.2"E
S3	Bortalav Lake	21°44'55.7"N 72°07'12.2"E
S4	Somnath	20°53'03.4"N 70°24'17.1"E
S5	Nageshwar Rd.	22°19'47.9"N 69°04'47.1"E
S6	Madhavpur	21°15'21.6"N 69°57'22.0"E
S7	Gangeshwar	20°42'19.3"N 70°57'24.1"E
S8	Sunset point Dwarka	22°14'39.8"N 68°57'16.9"E
S9	Near Rukmani temple	22°15'26.7"N 68°57'50.7"E
S10	Okha Road	22°26'23.0"N 69°02'45.7"E
S11	Kesaria road	20°46'09.6"N 70°56'17.2"E

Fig. S1 Sampling location of pigmented bacterial isolates screened for zeaxanthin production
